# Could reasons for admission help to screen unhealthy alcohol use in emergency departments? A multicenter French study

**DOI:** 10.3389/fpsyt.2023.1271076

**Published:** 2023-11-30

**Authors:** Jonathan Chabert, Céline Lambert, Julien Cabé, Cheryl J. Cherpitel, Benjamin Rolland, Farès Moustafa, Patrick Lesage, Delphine Ragonnet, Julie Geneste, Emmanuel Poulet, Maurice Dematteis, Mickael Naassila, Maryline Chalmeton, Pierre-Michel Llorca, Bruno Pereira, Ingrid De Chazeron, Georges Brousse

**Affiliations:** ^1^Service de Psychiatrie Adulte et d’Addictologie, CHU Clermont-Ferrand, CNRS, Université Clermont-Auvergne, Institut Pascal, Clermont-Ferrand, France; ^2^Unité de Biostatistiques, DRCI, CHU Clermont-Ferrand, Clermont-Ferrand, France; ^3^Alcohol Research Group, Emeryville, CA, United States; ^4^Service Universitaire d'Addictologie de Lyon, Centre Hospitalier Le Vinatier, Hospices Civils de Lyon et Université de Lyon, Lyon, France; ^5^Université Clermont Auvergne, INRAE, UNH, Clermont-Ferrand, France; ^6^Centre Hospitalier Métropole Savoie, Service des Urgences, Chambéry, France; ^7^Service Universitaire d’Addictologie de Lyon, Groupement Hospitalier Centre, Hospices Civils de Lyon, Lyon, France; ^8^Psychiatrie des Urgences - Groupement Hospitalier Edouard Herriot, EA 4615 « SIPAD », Université Lyon 1 - CH Le Vinatier, Lyon, France; ^9^Service Universitaire de Pharmaco-Addictologie, CHU Grenoble Alpes, Université Grenoble Alpes, Grenoble, France; ^10^INSERM UMRS1247-GRAP, Université Picardie Jules Verne, Amiens, France

**Keywords:** alcohol drinking, alcohol-related complications, heavy episodic drinking, emergency department, alcohol use disorder, mental disorder

## Abstract

**Background:**

Many patients admitted to general emergency departments (EDs) have a pattern of drinking that could lead to future alcohol-related complications. However, it is often difficult to screen these patients in the context of emergency. The aim of this study is to analyze whether reasons for admission could help to screen patients who have an unhealthy alcohol use.

**Method:**

Patients were recruited among six public hospital ED in France, between 2012 and 2014. During a one-month period in each hospital, anonymous questionnaires including sociodemographic questions, AUDIT-C and RAPS4-QF were administered to each patients visiting the ED. The reason for admission of each patient was noted at the end of their questionnaire by the ED practitioner.

**Results:**

Ten thousand Four hundred twenty-one patients were included in the analysis. Patients who came to the ED for injuries and mental disorders were more likely to report unhealthy alcohol use than non-harmful use or no use. Among male patients under 65 years old admitted to the ED for a mental disorder, 24.2% drank more than four drinks (40 g ethanol) in typical day at least four time a week in the last 12 months. Among these patients, 79.7% reported daily or almost daily heavy episodic drinking (HED, 60 g ethanol), and all were positive on the RAPS4-QF.

**Conclusion:**

This study highlights that unhealthy alcohol use is frequent among ED patients and particularly among those who come for injuries or mental disorders. Men under 65 years old with a mental disorder require special attention because of their increased prevalence of daily or almost daily HED.

## 1 Introduction

Alcohol is the psychoactive substance most used in France and a major public health problem since it is the leading cause of hospitalization in France (1) and is responsible for approximately 41,000 premature deaths each year (2). While many users will not be harmed by their consumption during their life, many others will therefore have alcohol-induced or related complications that could lead to functional impairment and in some cases to death (3).

In this context, screening for patients whose alcohol use is at-risk for or has already resulted in complications, also called unhealthy alcohol use, seems to be essential. However, while each country has developed its own guidelines to define what constitutes unhealthy alcohol use, there is currently no international consensus on permissible thresholds for alcohol quantity and frequency. For example, the National Institute of Alcohol Abuse and Alcoholism (NIAAA) recommends drinking in moderation by limiting intake to 2 standard American drinks (28 g ethanol) or less in a day for men and 1 standard American drink (14 g ethanol) or less in a day for women (4), while Canada’s Guidance recommends 1 or 2 standard Canadian drink (14 g ethanol) per week to avoid alcohol-related consequences (5). The Alcohol Use Disorders Identification Test – Consumption (AUDIT-C) (6) is a brief and democratized screening test which allows to screen unhealthy alcohol use according to the NIAAA guidelines. AUDIT-C has good sensitivity and specificity in primary care (7) and is also brief with only three questions: frequency of use, quantity and presence of heavy episodic drinking (HED), which is a pattern of drinking corresponding to the consumption of 60 or more grams of pure alcohol in a single occasion in the last month (8). AUDIT-C is however not very good to identify alcohol use disorder (AUD), and it is therefore recommended to use another questionnaire, such as the Rapid Alcohol Problems Screen 4–Quantity and Frequency (RAPS4-QF), to screen AUD. The RAPS4-QF is a six items quick self-administrated questionnaire, which has a sensitivity of 90.3% and a specificity of 84.1% for AUD (9). Some studies have even shown that the RAPS4-QF has a better sensitivity for detecting alcohol dependence than two other frequently used questionnaires: AUDIT and CAGE (10, 11). Nevertheless, despite the three questions of AUDIT-C and the six questions of RAPS4-QF, these tools are still considered too long to be incorporated into medical interviews in some time-sensitive settings, such as emergency departments (ED). Thus, many EDs practitioners do not evaluate alcohol consumption of their patients if alcohol is not related to their diagnostic or treatment, although a high prevalence of unhealthy alcohol use (almost 10%) has been found in EDs (12, 13). Furthermore, patients are significantly more likely to report heavy drinking, consequences of drinking or alcohol dependence in EDs than in medical offices (14), and as D’Onofrio et al. highlighted: “*patients are often more receptive to education in the moment of crisis*” (15). Thus, the ED could be an excellent site for screening patients for their alcohol use.

In 1996, Smith et al. proposed a quick scale, named the Paddington Alcohol Test (PAT), which is adapted to the ED context since it is modelled on the diagnostic approach of emergency physicians (16). The PAT, in its last version, lists the ten most prevalent presenting conditions of patients misusing alcohol (17). These presenting conditions are not specific diseases, but are combinations of symptoms, syndromes, or diseases, allowing the clinician to quickly obtain an overview of the patient’s probable alcohol consumption before having an exact diagnosis. The PAT was the alcohol screening tool most used in the United Kingdom since 41% of EDs using it in 2013 (18). This approach is quite rare in the literature, however. While many authors have focused on the relationship between alcohol consumption and some conditions frequently seen in the EDs, as falls (19–21) and injuries (22, 23), very few have evaluated whether reasons for admission could be an indicator of interest for alcohol screening.

Thus, the aim of our study is to determine whether certain reasons for admission are more frequently associated with unhealthy alcohol use. However, as unhealthy alcohol use is a broad and heterogeneous category of patients, comprising drinking patterns with different risks of developing alcohol-related complications or the type of complications (3), we then analyzed the association between certain drinking patterns and reason for ED visit.

## 2 Method

### 2.1 Study design and participants

Subjects were recruited in the ED of six public hospitals in Auvergne-Rhône-Alpes region (France), between 2012 and 2014. Anonymous questionnaires were given during one month period at each hospital, to those admitted to the ED and meeting inclusion criteria. All the participants gave their informed consent. We used a cluster random sampling design and area sampling method. Clusters were defined along geographic areas, and the month of recruiting was randomly assigned. All patients admitted in each ED unit over the course of one month were defined as a single cluster.

Inclusion criteria were aged 16 years and older, agreeing to participate, and speaking French. Patients unable to participate due to somatic or cognitive major impairments were excluded.

The protocol was approved by the Ethic Committee of Clinical Investigation Center of Auvergne-Rhône-Alpes inter-region in France (CE-CIC/GREN/12/09).

Complete study protocol has been published elsewhere (24).

### 2.2 Data collection

The questionnaire included sociodemographic data (age, gender…) and two self-administered alcohol screening tests, the AUDIT-C and the RAPS4-QF.

After completing the questionnaires, EDs staff completed the survey with the reason for admission of the patient in a predefined list inspired of the PAT list. When the reason for admission of the patient did not correspond to the presenting condition of the predefined list, the ED staff wrote the exact diagnosis in a box called “other.”

The eight presenting conditions listed were: “fall,” “assault,” “head injury,” “collapse,” “digestive complaint,” “chest tightness and/or palpitations,” “mental disorder” and “unwell” (suffering of unknown or imprecise origin).

### 2.3 Psychometric scales

The AUDIT-C is a scale evaluating alcohol drinking with three questions: “How often did you have a drink containing alcohol in the past year?,” “How many drinks did you have on a typical day when you were drinking in the past year?” and “How often did you have six or more drinks on one occasion in the past year?.” For each question, a standard drink corresponds to 10 g of ethanol. Each answer is rated between 0 to 4, so the maximum overall score is 12. A score ≥ 4 for men or ≥ 3 for women suggests an unhealthy alcohol use according to NIAAA.

The RAPS4-QF consists of the four questions of RAPS4: “During the last year, have you had a feeling of guilt or remorse after drinking? (Remorse),” “During the last year, has a friend or family member ever told you about things you said or did while you were drinking that you could not remember? (Amnesia, also called Blackouts),” “During the last year, have you failed to do what was normally expected from you because of drinking? (Perform),” “Do you sometimes take a drink in the morning, when you first get up? (Starter, also called eye opener),” and two additional questions on quantity and frequency: “During the last year, have you had five or more drinks on at least one occasion? (Quantity),” “During the last year, do you drink as often as once a month? (Frequency).” A positive response on any one of the RAPS4 items and/or both of the QF items is considered positive on the RAPS4-QF and suggests AUD.

### 2.4 Categories of conditions for analysis

The reason for admission of patients was classified into 23 categories. The first eight categories were the eight presenting conditions listed (see above). The other 15 categories were partially based on the International Classification of Disease 11^th^ revision for Mortality and Morbidity Statistics (ICD-11 MMS). Patients who came to the EDs for obstetrical or dental conditions were excluded because there are specific EDs in France which treat these conditions, and therefore the few patients in these study with those conditions were likely not representative.

For the first stage of the analysis, based on the AUDIT-C, patients were divided into three categories: “no use,” which correspond to ED’s patients who did not drink alcohol the last year, “low risk alcohol use,” which correspond to ED patients whose alcohol use met NIAAA guidelines, and “unhealthy alcohol use,” which correspond to ED patients whose alcohol use is above NIAAA guidelines.

**Figure 1:**
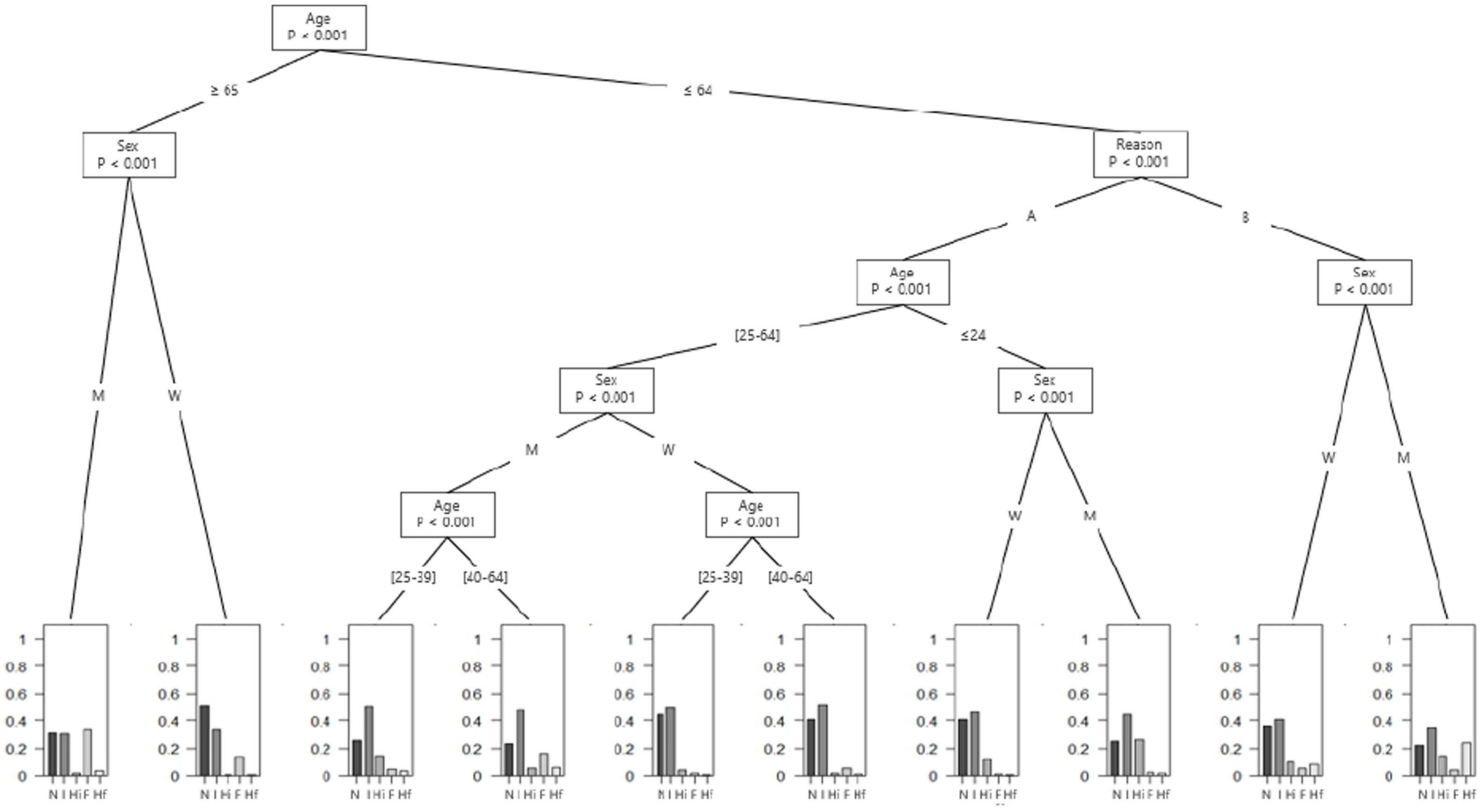
Amount and frequency of alcohol use among emergency department’s patients according to their reason of admission, age and gender. A = “Fall,” “Assault,” “Head injury,” “Collapse,” “Digestive complaint,” “Chest tightness and/or palpitations,” “Unwell,” “Public road accident,” “Medical examination/Post-acute care,” “Dermatological pathologies,” “Altered general condition,” “Infectious diseases,” “Injury, poisoning or certain other consequences of external causes,” “Endocrine, nutritional or metabolic diseases,” “Pathologies of the circulatory system,” “Pathologies of the respiratory system,” “Nervous pathologies,” “Pathologies of the visual system,” “Otolaryngology pathologies,” “Osteoarticular pathologies,” “Genitourinary pathologies” and “Pathologies of blood”; B = “Mental disorder”; F = frequent drinking; Hf = heavy frequent drinking; Hi = heavy infrequent drinking; I = infrequent drinking; M = Men; N = Non-drinker; W = Women.

**Figure 2:**
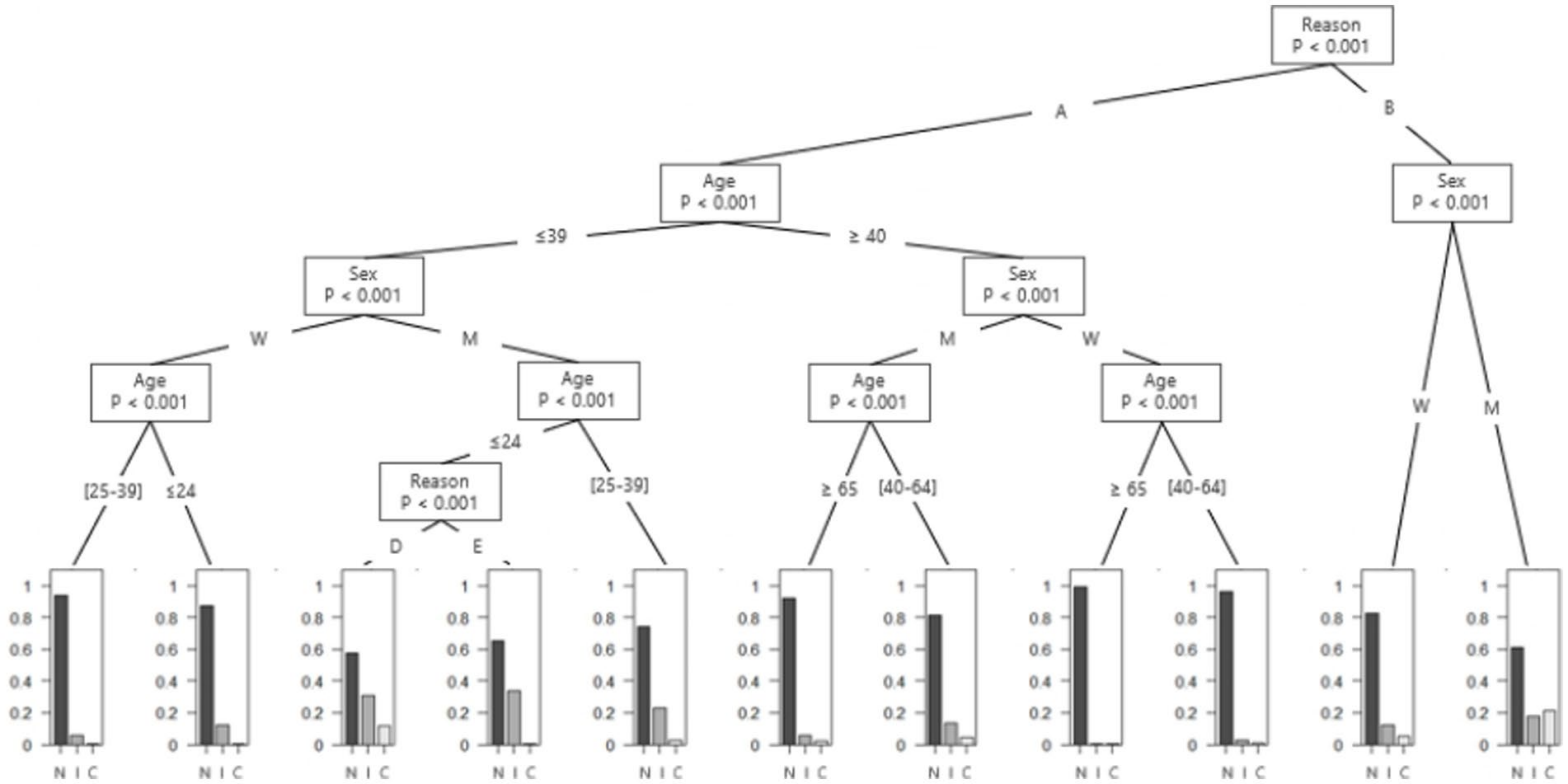
Proportion of heavy episodic drinking among emergency department’s patients according to their reason of admission, age and gender. A = “Fall,” “Assault,” “Head injury,” “Collapse,” “Digestive complaint,” “Chest tightness and/or palpitations,” “Unwell,” “Public road accident,” “Medical examination/Post-acute care,” “Dermatological pathologies,” “Altered general condition,” “Infectious diseases,” “Injury, poisoning or certain other consequences of external causes,” “Endocrine, nutritional or metabolic diseases,” “Pathologies of the circulatory system,” “Pathologies of the respiratory system,” “Nervous pathologies,” “Pathologies of the visual system,” “Otolaryngology pathologies,” “Osteoarticular pathologies,” “Genitourinary pathologies” and “Pathologies of blood”; B = “Mental disorder”; C = chronic heavy episodic drinking, D = “Pathologies of the respiratory system” and “Otolaryngology pathologies”; E = “Fall,” “Assault,” “Head injury” “Collapse,” “Gastro-intestinal,” “Chest tightness and/or palpitation,” “Unwell,” “Public road accident,” “Medical examination/Post-acute care,” “Dermatological pathologies,” “Infectious diseases,” “Injury, poisoning or certain other consequences of external causes,” “Pathologies of the circulatory system,” “Nervous pathologies,” “Pathologies of the visual system,” “Osteoarticular pathologies,” “Genitourinary pathologies” and “Pathologies of blood”; I = irregular heavy episodic drinking; M = Men; N = no heavy episodic drinking; W = Women.

For the second stage of the analysis, as the unhealthy alcohol use is a heterogeneous category that presents a broad spectrum of risk of complications for patients, we decided to explore more specific pattern of alcohol use to gain precision. The first pattern is based on amount and frequency of alcohol use, and use the two first questions of the AUDIT-C to divide the patients into five categories: “non-drinker,” “infrequent drinker” (those who consume less than four times a week and four or less standard drinks in a typical day), “heavy infrequent drinker” (those who consume less than four times a week and more than four standard drinks in a typical day), “frequent drinker” (those who consume four or more times a week and four or less standard drinks in a typical day) and “heavy frequent drinker” (those who consume more than four times a week and more than four standard drinks in a typical day). The quantity cut-off of four standard drinks (40 g ethanol) was chosen because it synthetized both the four-drink-per-occasion limit proposed by the WHO Collaborative group (25), and the NIAAA guidelines that consider drinking more than three standard American drinks (42 g ethanol, about four standard French drinks) for women and four standard American drinks for men (56 g ethanol, about five standard French drinks) is heavy drinking (26). This threshold has also been used elsewhere as cut-off for heavy drinking (3, 27). The drinking frequency cut-off was used to differentiate non-daily drinkers from almost daily-drinkers, since daily alcohol drinking without an alcohol free day increase risk of several complications (28). This is also why many guidelines recommend one or more alcohol-free days per week (4, 8, 29). From these categories, we proposed two conditional inference trees, one based on the amount and frequency of alcohol consumption to assess heavy drinking (Figure 1), and the other based on the presence of HED (Figure 2). Conditional inference trees have the advantage of providing outcomes that can be easily used by practitioners when interviewing patients.

For the tree based on the presence of HED, the third question of the AUDIT-C was used to divide patients into three categories: “No HED,” “HED” and “Chronic HED.” The first cut-off is based on the WHO’s definition of HED (“at least once a month” (8)), and the second cut-off is used to differentiate irregular HED from daily or almost daily heavy drinking (3).

### 2.5 Statistical analysis

Statistical analysis was performed using Stata software (version 15; StataCorp, College Station, Texas, United States) and R (version 4.1.3; R Foundation for Statistical Computing, Vienna, Austria). All tests were two-sided, with an alpha level set at 5%. No correction for multiple testing was applied in the analysis of outcomes or subgroup analysis (30). The patients’ age was presented as mean ± standard deviation, and the categorical variables as the number of patients, associated percentages, and 95% confidence interval (CI). Comparisons between included and non-included patients were made by the Student’s t test for the patients’ age, and by the Chi-squared test for categorical variables (data not shown). Comparisons between independent groups (e.g., no use vs. low risk alcohol use vs. unhealthy alcohol use) were made by ANOVA for the patients’ age and by the Chi-squared test for categorical variables. When appropriate (omnibus value of p less than 0.05), a Tukey–Kramer test was performed after ANOVA, and a Marascuilo *post-hoc* procedure after Chi-squared test. Effect sizes were measured by Cramér’s V and interpreted as: 0.1 = small effect, 0.3 = medium effect and 0.5 = large effect. Then, conditional inference trees were built with the “party” R package (31). The tree begins with a node called the root, representing the entire training dataset. At each node of the tree, the input variable with the strongest association to the response is recursively selected to split the data binary into two subsets. The splitting process is repeated iteratively, choosing at each step the variable that optimizes the splitting criterion. Thus, each variable (e.g., age, gender, …) can be split at different stages of the analysis. Tree construction continues until a stopping criterion is met, such as reaching a maximum tree depth or a minimum number of samples per subsets. Two regressions tree were built with the same input variables (age in four categories, gender, and reasons for admission) but with distinct responses: the first based on the amount and frequency of alcohol consumption, and the second based on the presence of HED. The four categories for age were selected in advance. As these analyses were only descriptive, the entire sample was used (no validation set).

## 3 Results

Among the 23,834 patients admitted to one of the six EDs during the study period, 11,632 patients filled out the questionnaires, and 10,421 patients were included in the analysis. The excluded patients were those with obstetrical or dental conditions and patients with significant missing data on age or gender. See Supplementary Table 1 for details on the included population.

### 3.1 No use vs. low risk alcohol use vs. unhealthy alcohol use

Similar proportion of patients were in each of the three categories: 34.3% for no use, 29.9% for low-risk alcohol use and 35.8% for unhealthy alcohol use. See Table 1 for the comparison of sociodemographic data between the three categories.

**Table 1:** Sociodemographic characteristics of emergency department’s patients according to their alcohol use.

	Overall (*N* = 10,421)	No use (*N* = 3,576)	Low risk alcohol use (*N* = 3,111)	Unhealthy alcohol use (*N* = 3,734)	*p*-value	*V*
Age (years)	46.7 ± 21.7	48.7 ± 23.0	46.9 ± 20.5	44.6 ± 21.3	**<0.001**	−
Female gender	4,734 (45.4)	2,094 (58.6)	1,446 (46.5)	1,194 (32.0)	**<0.001**	0.22
Having children*	6,288 (60.6)	2,345 (65.9)	1,940 (62.6)	2,003 (53.9)	**<0.001**	0.11
Marital status†					**<0.001**	0.13
Single	3,565 (34.4)	1,089 (30.6)	942 (30.5)	1,534 (41.3)		
In relationship	1,239 (12.0)	254 (7.1)	447 (14.5)	538 (14.5)		
Married	3,793 (36.7)	1,487 (41.9)	1,192 (38.6)	1,114 (30.0)		
Divorced	935 (9.0)	322 (9.1)	274 (8.9)	339 (9.1)		
Widowed	818 (7.9)	401 (11.3)	230 (7.5)	187 (5.0)		
Education level‡					**<0.001**	0.18
None	2,554 (24.9)	1,351 (38.4)	572 (18.6)	631 (17.2)		
Secondary education	4,904 (47.7)	1,572 (44.7)	1,505 (48.9)	1,827 (49.7)		
Bachelor’s degree	1,828 (17.8)	450 (12.9)	613 (19.9)	765 (20.8)		
Master’s degree	787 (7.7)	111 (3.2)	305 (9.9)	371 (10.1)		
Doctorate’s degree	197 (1.9)	31 (0.9)	81 (2.6)	85 (2.3)		

Data are presented as number of patients (percentages in columns), or as mean ± standard deviation.

V: Cramér’s V in absolute value (interpreted as: 0.1 = small effect, 0.3 = medium effect and 0.5 = large effect).

* The number of available data was 10,374 (overall), 3,557 (no use), 3,099 (low risk alcohol use), and 3,718 (unhealthy alcohol use).

† The number of available data was 10,350 (overall), 3,553 (no use), 3,085 (low risk alcohol use), and 3,712 (unhealthy alcohol use).

‡ The number of available data was 10,270 (overall), 3,515 (no use), 3,076 (low risk alcohol use), and 3,679 (unhealthy alcohol use).

Table 2 describes the proportion of alcohol drinking for each reason for admission. Patients who presented to ED for (ranking from the highest to the lowest percentage): “head injury,” “mental disorder,” “assault,” “injury, poisoning or certain other consequences of external causes,” “fall,” “public road accident,” “pathologies of the circulatory system,” “chest tightness and/or palpitations,” “medical examination/post-acute care” or “genitourinary pathologies,” were more likely to engage in unhealthy drinking than low-risk drinking, or not to drink at all.

**Table 2:** Reasons for admission of emergency department’s patients according to their alcohol use.

	No use (*N* = 3,576)^1^	95% CI^2^	Low risk alcohol use (*N* = 3,111)^1^	95% CI^2^	Unhealthy alcohol use (*N* = 3,734)^1^	95% CI^2^
Fall	485 (31.4)	29, 34%	454 (29.4)	27, 32%	604 (39.1)	37, 42%
Assault	89 (34.2)	29, 40%	60 (23.1)	18, 29%	111 (42.7)	37, 49%
Head injury	63 (23.2)	18, 29%	68 (25.0)	20, 31%	141 (51.8)	46, 58%
Collapse	266 (37.6)	34, 41%	231 (32.6)	29, 36%	211 (29.8)	26, 33%
Digestive complaint	485 (41.1)	38, 44%	374 (31.7)	29, 34%	320 (27.1)	25, 30%
Chest tightness and/or palpitations	191 (33.7)	30, 38%	167 (29.5)	26, 33%	208 (36.7)	33, 41%
Mental disorder	223 (30.8)	27, 34%	172 (23.8)	21, 27%	329 (45.4)	42, 49%
Unwell	255 (45.3)	41, 50%	157 (27.9)	24, 32%	151 (26.8)	23, 31%
Public road accident	84 (25.2)	21, 30%	119 (35.7)	31, 41%	130 (39.0)	34, 45%
Medical examination/Post-acute care	42 (32.6)	25, 41%	42 (32.6)	25, 41%	45 (34.9)	27, 44%
Dermatological pathologies	102 (37.2)	32, 43%	83 (30.3)	25, 36%	89 (32.5)	27, 38%
Altered general condition	53 (50.0)	41, 59%	23 (21.7)	15, 31%	30 (28.3)	20, 38%
Infectious diseases	60 (37.5)	30, 46%	49 (30.6)	24, 38%	51 (31.9)	25, 40%
Injury, poisoning or certain other consequences of external causes	419 (26.4)	24, 29%	499 (31.5)	29, 34%	667 (42.1)	40, 45%
Endocrine, nutritional or metabolic diseases	29 (42.6)	31, 55%	24 (35.3)	24, 48%	15 (22.1)	13, 34%
Pathologies of the circulatory system	65 (31.2)	25, 38%	65 (31.2)	25, 38%	78 (37.5)	31, 44%
Pathologies of the respiratory system	147 (45.1)	40, 51%	73 (22.4)	18, 27%	106 (32.5)	28, 38%
Nervous pathologies	172 (37.2)	33, 42%	156 (33.8)	29, 38%	134 (29.0)	25, 33%
Pathologies of the visual system	35 (22.6)	16, 30%	69 (44.5)	37, 53%	51 (32.9)	26, 41%
Otolaryngology pathologies	56 (47.1)	38, 56%	25 (21.0)	14, 30%	38 (31.9)	24, 41%
Osteoarticular pathologies	144 (38.9)	34, 44%	104 (28.1)	24, 33%	122 (33.0)	28, 38%
Genitourinary pathologies	92 (33.9)	28, 40%	85 (31.4)	26, 37%	94 (34.7)	29, 41%
Pathologies of blood	19 (47.5)	32, 64%	12 (30.0)	17, 47%	9 (22.5)	11, 39%

1 Data are presented as number of patients (percentages in rows).

2 CI is confidence interval.

### 3.2 Regression trees

#### 3.2.1 First tree – amount and frequency of alcohol use

The first tree describes the amount and frequency of alcohol use in ED patients according to reasons for admission, gender, and age (Figure 1). A first distinction is made between patients aged 65 years old and over and patients aged less than 65 years old.

In patients aged 65 years old and over, regardless of gender, less than 2.5% drank more than four drinks a typical day.

In patients aged less than 65 years old, there is an important distinction in alcohol drinking depending on the reason for admission and gender. There was a significant difference in the proportion of heavy frequent drinkers (more than four drinks in a typical day at least four times a week during the last 12 months) between men under 65 years old who came to the ED for a reason related to a mental disorder (24.2, 95%CI: 19.5 to 29.4%), women under 65 years old who came for a reason related to a mental disorder (8.3, 95%CI: 5.6 to 11.8%) and all others patients (2.3, 95%CI: 2.0 to 2.6%) (*p* < 0.001).

#### 3.2.2 Second tree – heavy episodic drinking

The second tree describes HED in ED’s patients according to reasons for admission, gender, and age (Figure 2). A first distinction is made according to the reason for admission, with patients who came for reasons related to a mental disorder on the one hand and patients who came for other reasons on the other.

Among patients who came for a reason related to a mental disorder, the proportion of chronic HED in the last 12 months was 21.3% (95%CI: 17.1 to 26.1%) for men and 5.2% (95%CI: 3.2 to 7.9%) for women. The proportion of irregular HED was 17.8% (95%CI: 13.8 to 22.3%) for men and 12.2% (95%CI: 9.1 to 15.9%) for women.

Among patients who did not come for a reason related to a mental disorder, there was a low proportion of HED for men aged 65 years old and over and women aged 40 years and over (less than 10%). However, for men between 25 and 39 years old, the proportion of irregular HED was 22.7% (95%CI: 20.6 to 24.9%).

Among male patients younger than 25 years old, who were admitted for any condition other than “Mental disorder,” “Altered General Condition,” “Endocrine, nutritional or metabolic diseases,” “Pathologies of the respiratory system” or “Otolaryngology pathologies,” the proportion or irregular HED in the last 12 months was 33.8% (95%CI: 30.9 to 36.7%). Among those with respiratory or otolaryngology conditions the proportion of irregular HED was 30.8% (95%CI: 14.3 to 51.8%) and chronic HED was 11.5% (95%CI: 2.4 to 30.2%).

### 3.3 Heavy frequent drinkers among men under the age of 65 who came for a reason related to a mental disorder

As described above, 24.2% of men under age 65 years who came for a reason related to a mental disorder were heavy frequent drinker. The proportion of chronic HED in these patients was 79.7% and the proportion of irregular HED 16.2%. All these patients were positive to the RAPS4-QF, and the majority (56.8%) drank at least 10 drinks in a typical day. Table 3 shows characteristics of these individuals.

**Table 3:** Heavy frequent drinkers among men under the age of 65 who came for a reason for admission related to a mental disorder (MMD).

	Overall (*N* = 10,421)	MMD (*N* = 74)	Others (*N* = 10,347)	*p*-value	*V*
*Age (years)*					
Having children*	6,288 (60.6)	42 (56.8)	6,246 (60.6)	0.50	0.007
Marital status^†^				**<0.001**	0.05
Single	3,565 (34.4)	42 (56.8)	3,523 (34.3)		
In relationship	1,239 (12.0)	8 (10.8)	1,231 (12.0)		
Married	3,793 (36.6)	10 (13.5)	3,783 (36.8)		
Divorced	935 (9.0)	12 (16.2)	923 (9.0)		
Widowed	818 (7.9)	2 (2.7)	816 (7.9)		
Education level^‡^				0.21	0.02
None	2,554 (24.9)	15 (20.5)	2,539 (24.9)		
Secondary education	4,904 (47.8)	42 (57.5)	4,862 (47.7)		
Bachelor’s degree	1,828 (17.8)	8 (11.0)	1,820 (17.8)		
Master’s degree	787 (7.7)	5 (6.8)	782 (7.7)		
Doctorate’s degree	197 (1.9)	3 (4.1)	194 (1.9)		
Heavy episodic drinking				**<0.001**	0.42
None	8,852 (84.9)	3 (4.1)	8,849 (85.5)		
Irregular heavy episodic drinking	1,305 (12.5)	12 (16.2)	1,293 (12.5)		
Chronic heavy episodic drinking	264 (2.5)	59 (79.7)	205 (2.0)		
Alcohol amounts a typical day#				**<0.001**	0.30
1 to 2 drinks	4,120 (60.2)	0 (0.0)	4,120 (60.8)		
3 to 4 drinks	1,555 (22.7)	0 (0.0)	1,555 (23.0)		
5 to 6 drinks	623 (9.1)	16 (21.6)	607 (9.0)		
7 to 9 drinks	250 (3.7)	16 (21.6)	234 (3.5)		
≥10 drinks	297 (4.3)	42 (56.8)	255 (3.8)		
Positive RAPS4-QF	3,130 (30.0)	74 (100)	3,056 (29.5)	**<0.001**	0.13

Data are presented as number of patients (percentages in columns), or as mean ± standard deviation. MMD: heavy frequent drinkers among men under the age of 65 who came for a reason for admission related to a mental disorder; RAPS4-QF: rapid alcohol problems screen 4-quantity and frequency; V: Cramér’s V in absolute value (interpreted as: 0.1 = small effect, 0.3 = medium effect and 0.5 = large effect).

* The number of available data was 10,374 (overall), 74 (MMD), and 10,300 (others).

† The number of available data was 10,350 (overall), 74 (MMD), and 10,276 (others).

‡ The number of available data was 10,270 (overall), 73 (MMD), and 10,197 (others).

# he number of available data was 6,845 (overall), 74 (MMD), and 6,771 (others).

## 4 Discussion

This study aimed to determine whether certain reasons for admission are more frequently associated with unhealthy alcohol use, according to NIAAA guidelines. We saw that there is no reason for admission that would allow practitioners to not screen for unhealthy alcohol use, since even for patients who go to the ED for endocrine, nutritional, or metabolic diseases, which are the reasons for the lowest rates of unhealthy alcohol use, more than one in five had unhealthy alcohol use. Furthermore, there were some reasons for admission for which patients were more likely to have unhealthy drinking than low risk drinking or not to drink at all. Finally, 24% of men, under 65, who came for a mental disorder in ED have a heavy alcohol use, more than four drinks in a typical day, more than four days a week in the last 12 months. Of these patients, 80% had daily or almost daily HED, and 16% had HED at least once a month.

ED’s practitioners must be particularly attentive to two categories of reasons for admission: injuries and mental disorder. In this study, at least 39% of patients who came for mental disorders, or an injury were classified in the unhealthy alcohol use category according to the AUDIT-C. The relationship between alcohol drinking and injuries is well known. Some authors found that about one third of patients admitted to the ED for injury reported drinking prior to the event (22, 32–34). This might suggest that blood alcohol concentration (BAC) or breath alcohol concentration (BrAC) could be enough to detect patients with an unhealthy alcohol use in the ED. Indeed, there is a correlation between alcohol use and risk of injury, with, according to Cherpitel et al., risk doubling with the first drink and continuing to increase with subsequent drinks (35). However, alcohol-related injuries are not limited to acute alcohol intoxication. Some damages caused by long term alcohol use, such as alcohol neuropathy (36), cerebellar impairment (37, 38), etc., can also increase the risk of injuries. These other risk factors could explain that 39% of our patients who fell had an unhealthy alcohol use, while two other studies with large samples found that only 2% of patients who came to the ED for a fall had drunk before injury (20, 21). Thus, to focus solely on BrAC and BAC could lead to missing many patients with an unhealthy alcohol use. This conclusion was also highlighted by Browne et al. who found that 30% of patients with an AUD in Australia were not identified by ED staff as having alcohol-related injury or an alcohol related problem (39).

Apart from injury, practitioners must be attentive to patients who come to the ED for a complaint related to a mental disorder. Our data suggest that almost 7% of ED patients came for a mental disorder, which is more frequent than the average of other countries (4%) (40), and that 45% of them had an AUDIT-C score in favor of unhealthy alcohol use. This high rate of unhealthy alcohol use is not surprising as AUD is highly associated with many mental disorders (41–44). This study was designed to provide an overview of alcohol use associated with the general reason for ED admission and does not allow us to discern which mental disorder(s) are most closely associated with unhealthy alcohol use. This information could prove valuable to better screen patients, especially since the literature on this subject is relatively thin. Among the few studies reported, 33% of schizophrenic or bipolar patients and 22% of depressive or anxious patients who came to psychiatric emergency had at least hazardous drinking (45).

Although, we cannot describe which mental disorder is more closely associated with unhealthy alcohol use, data here suggest that there is a difference in unhealthy alcohol use’s severity according to gender and age. The regression tree showed that men under age 65 admitted to the ED with a mental disorder had a 24% risk of drinking more than four standard drinks in a typical day, at least four times a week during the last 12 months. Such high frequency and amount of alcohol use can lead to many diseases. Indeed, it is well known that the risk of developing certain diseases increases linearly, or even exponentially, with alcohol use (3). Furthermore, alcohol use can destabilize the course of an underlying mental illness. For example, alcohol use in bipolar patients leads to more mood swings (46), more rapid cycling (46–48), and a higher suicidal risk (47–50). In depression, alcohol use is associated with a higher level of depressive symptoms (51) and a more altered quality of life (52). Moreover, we found that 80% of heavy frequent drinker admitted to the ED with a mental disorder had daily or almost daily HED and all of them were positive on RAPS4-QF. Thus, in addition to consequences related to alcohol frequency and amount of consumption, these patients have HED-related risks, such as an increased risk of coronary heart diseases (53), high blood pressure (53), sexually transmitted diseases (54, 55), and injuries (56). Furthermore, the RAPS4-QF is an instrument with 90% sensitivity and 84% specificity for screening for DSM-5 AUD. This suggest that these patients not only have an unhealthy alcohol use but also have an AUD. This is significantly higher than the rest of the sample which was 30% RAPS4-QF positive, a percentage relatively similar to that found elsewhere. (57).

Our study has some limitations. First, we used self-reported drinking to measure alcohol use, which is a common method with an average validity of 92% (58), but which could be less accurate for frequent heavy drinking individuals (59). However, in analysis here, patients who drank more than four drinks in a typical day were grouped into one category, and while it is possible that those who drink the most may report with less accuracy the actual number of drinks they consume, it would be surprising if they reported less than four drinks. Secondly, we used broad categories of reasons for admission, which can lead to a loss of information. For example, it is likely that if the gastro-intestinal category has been further delineated, some reasons for admission would have been more strongly related to unhealthy alcohol use (liver diseases). However, the aim of our study was not to describe which pathology is associated with alcohol use, because the association of alcohol with many diseases is already known (3). Our aim was to propose “red flags” for ED’s staff to help them to quickly screen for unhealthy alcohol use based admission complaint. Third, all the patients with a reason for admission related to the management of an alcohol intoxication or an alcohol withdrawal syndrome were classified in the mental disorder category. This logically raises the question of whether the high prevalence of daily or almost daily HED in this category was not primarily due to these patients, and whether it is necessary to be so concerned about alcohol use for other mental disorders. We do not have the necessary data to answer this question, but it should be noted that 45% of the patients whose reason for admission was a mental disorder had an unhealthy alcohol use, which represents considerably more patients than those admitted to the ED for a withdrawal syndrome or alcohol intoxication. Furthermore, even if the heaviest drinkers were mainly patients who came for withdrawal syndrome or alcohol intoxication, the message from our data would also be interesting. Indeed, it would mean that, in ED, an acute intoxication and a withdrawal syndrome should never be considered as an acute phenomenon, but as the emerging part of a deeper problem since many of these patients have daily or almost daily HED. Fourth, we recruited patients during one month in each ED and, although each month was randomly chosen, the study took place mainly during summer, and it is possible that there may be seasonal variation in findings here. Finally, we included only patients able to answer questionnaires, it therefore did not represent all the ED’s patients.

To conclude, this study highlights that unhealthy alcohol use is frequent among ED patients and particularly among those who are admitted for injuries or mental disorders. Men under age 65 who present with a mental disorder require special attention because of their increased likelihood of reporting daily or almost daily HED.

## Data availability statement

The original contributions presented in the study are included in the article/Supplementary materials, further inquiries can be directed to the corresponding author/s.

## Ethics statement

The studies involving humans were approved by Comité d’Ethique des Centres d’Investigation Clinique de l’inter-région Rhône-Alpes-Auvergne. The studies were conducted in accordance with the local legislation and institutional requirements. Written informed consent for participation in this study was provided by the participants’ legal guardians/next of kin.

## Author contributions

JoC: Writing – original draft, Conceptualization, Data curation, Methodology, Software, Writing – review & editing. CL: Software, Validation, Writing – review & editing. JuC: Conceptualization, Methodology, Writing – review & editing. CC: Conceptualization, Writing – review & editing. BR: Conceptualization, Data curation, Writing – review & editing. FM: Conceptualization, Investigation, Methodology, Writing – review & editing. PL: Conceptualization, Investigation, Methodology, Writing – review & editing. DR: Conceptualization, Investigation, Methodology, Writing – review & editing. JG: Conceptualization, Funding acquisition, Investigation, Methodology, Writing – review & editing. EP: Conceptualization, Investigation, Methodology, Writing – review & editing. MD: Conceptualization, Investigation, Writing – review & editing. MN: Conceptualization, Investigation, Writing – review & editing. MC: Conceptualization, Data curation, Funding acquisition, Investigation, Methodology, Writing – review & editing. P-ML: Conceptualization, Funding acquisition, Investigation, Methodology, Writing – review & editing. BP: Software, Writing – review & editing. IC: Conceptualization, Data curation, Funding acquisition, Investigation, Methodology, Writing – review & editing. GB: Conceptualization, Data curation, Funding acquisition, Investigation, Methodology, Supervision, Writing – review & editing.
