# Supplementary material for: Could reasons for admission help to screen unhealthy alcohol use in emergency departments? A multicenter French study
Source: Front Psychiatry. 2023 Nov 30;14:1271076. doi: 10.3389/fpsyt.2023.1271076 (PMC10719849; doi:10.3389/fpsyt.2023.1271076)
Supplement: Supplementary file 1 [file Table_1.DOCX]

| **Supplementary table 1: Characteristics of emergency department’s patients.** | |
| --- | --- |
|  | **Overall**  **(N = 10,421)** |
| Age (years) | 46.7 ± 21.7 |
| Female gender | 4,734 (45.4) |
| Having children | 6,288 (60.3) |
| Marital status |  |
| Single | 3,565 (34.2) |
| In relationship | 1,239 (11.9) |
| Married | 3,793 (36.4) |
| Divorced | 935 (9.0) |
| Widowed | 818 (7.8) |
| Education level |  |
| None | 2,554 (24.5) |
| Secondary education | 4,904 (47.1) |
| Bachelor’s degree | 1,828 (17.5) |
| Master’s degree | 787 (7.6) |
| Doctorate’s degree | 197 (1.9) |
| Reason for admission |  |
| Fall | 1,543 (14.8) |
| Assault | 260 (2.5) |
| Head injury | 272 (2.6) |
| Collapse | 708 (6.8) |
| Digestive complaint | 1,179 (11.3) |
| Chest tightness and/or palpitations | 566 (5.4) |
| Mental disorder | 724 (6.9) |
| Unwell | 563 (5.4) |
| Public road accident | 333 (3.2) |
| Medical examination/Post-acute care | 129 (1.2) |
| Dermatological pathologies | 274 (2.6) |
| Altered general condition | 106 (1.0) |
| Infectious diseases | 160 (1.5) |
| Injury, poisoning or certain other consequences of external causes | 1,585 (15.2) |
| Endocrine, nutritional or metabolic diseases | 68 (0.7) |
| Pathologies of the circulatory system | 208 (2.0) |
| Pathologies of the respiratory system | 326 (3.1) |
| Nervous pathologies | 462 (4.4) |
| Pathologies of the visual system | 155 (1.5) |
| Otolaryngology pathologies | 119 (1.1) |
| Osteoarticular pathologies | 370 (3.6) |
| Genitourinary pathologies | 271 (2.6) |
| Pathologies of blood | 40 (0.4) |
| *Data are presented as number of patients (associated percentages), or as mean ± standard deviation.* | |
